# Dynamic Assay for Profiling Anti-SARS-CoV-2 Antibodies and Their ACE2/Spike RBD Neutralization Capacity

**DOI:** 10.3390/v13071371

**Published:** 2021-07-15

**Authors:** Thomas Phelan, Jean Dunne, Niall Conlon, Clíona Ní Cheallaigh, W. Mark Abbott, Raquel Faba-Rodriguez, Fatima Amanat, Florian Krammer, Mark A. Little, Gerry Hughes, Colm Bergin, Colm Kerr, Sudharshana Sundaresan, Aideen Long, William McCormack, Gareth Brady

**Affiliations:** 1 Department of Clinical Medicine, School of Medicine, Trinity Translational Medicine Institute, Trinity College Dublin, Dublin, D08 W9RT, Ireland; nicheacm@tcd.ie (C.N.C.); ghughes@stjames.ie (G.H.); cbergin@stjames.ie (C.B.); ckerr@stjames.ie (C.K.); ssundare@tcd.ie (S.S.); longai@tcd.ie (A.L.); mccormw@tcd.ie (W.M.); 2 Department of Immunology, St. James’s Hospital, D08 NHY1 Dublin, Ireland; dunnej1@tcd.ie (J.D.); niaconlon@stjames.ie (N.C.); 3 Department of Immunology, School of Medicine, Trinity College Dublin, D02 R590 Dublin, Ireland; 4 Department of Infectious Diseases, St James’s Hospital, D08 NHY1 Dublin, Ireland; 5 Peak Proteins Ltd., Alderley Park, Mereside, Macclesfield SK10 4TG, UK; mark.abbott@peakproteins.com (W.M.A.); raquel.faba@peakproteins.com (R.F.-R.); 6 Department of Microbiology, Icahn School of Medicine, Mount Sinai, New York, NY 10029-5674, USA; fatima.amanat@icahn.mssm.edu (F.A.); florian.krammer@mssm.edu (F.K.); 7 Trinity Health Kidney Centre, Trinity Translational Medicine Institute, Trinity College Dublin, St. James’ Hospital Campus, D08 W9RT Dublin, Ireland; mlittle@tcd.ie; 8 Department of Pharmacy, St James’s Hospital, D08 NHY1 Dublin, Ireland

**Keywords:** antibody, enzyme-linked immunosorbent assay, neutralization, SARS-CoV-2, serology, pseudovirus infection model

## Abstract

Serological assays have been widely employed during the coronavirus disease 2019 (COVID-19) pandemic to measure antibody responses to severe acute respiratory syndrome coronavirus 2 (SARS-CoV-2) and to track seroconversion in populations. However, currently available assays do not allow determination of neutralization capacity within the assay protocol. Furthermore, commercial serology assays have a high buy-in cost that is inaccessible for many research groups. We have replicated the serological enzyme-linked immunosorbent assay for the detection of SARS-CoV-2 antibody isotypes, developed at the Icahn School of Medicine at Mount Sinai, New York. Additionally, we have modified the protocol to include a neutralization assay with only a minor modification to this protocol. We used this assay to screen local COVID-19 patient sera (*n* = 91) and pre-COVID-19 control sera (*n* = 103), and obtained approximate parity with approved commercial anti-nucleoprotein-based assays with these sera. Furthermore, data from our neutralization assay closely aligns with that generated using a spike-based pseudovirus infection model when a subset of patient sera was analyzed.

## 1. Introduction

In December 2019, severe acute respiratory syndrome coronavirus 2 (SARS-CoV-2) was first identified in Wuhan, China [1]. As of the 10th of June 2021, there have been approximately 174 million confirmed cases of coronavirus disease 2019 (COVID-19), resulting in a staggering 3.75 million deaths worldwide [2].

At the time of writing, the FDA have listed 77 emergency use authorized serology tests on their website [3]. Most of these approved tests are chemiluminescent immunoassays which can be run on automated platforms and are capable of detecting isotype-specific antibodies. Enzyme-linked immunosorbent assay (ELISA)-based serology tests have also been approved, as well as lateral flow type assays. The predominant antigen used in these assays is the spike antigen (49 assays), both spike and nucleocapsid antigens are used in 18 of the approved tests (mainly lateral flow), with the remainder composed of nucleocapsid-based tests (eight tests), and one assay from United Biomedical is reported to detect spike, nucleocapsid and membrane protein.

Some of the initial tests released at the beginning of the pandemic had poor sensitivity and specificity values likely based on the poor quality of antigens used. The sensitivity, or ability to detect those with antibodies to SARS-CoV-2 (‘true positive rate’), and their specificity, or their ability to distinguish those with SARS-CoV-2 antibodies (‘true negative rate’), on the approved tests are generally high, with many tests showing near 100% specificity and high (>90%) sensitivity values.

In March 2020, Yan et al. reported that angiotensinogen converting enzyme (ACE2) is the human receptor for the spike protein of SARS-CoV-2 and determined a high resolution Cyro-EM structure for the complex [4]. There have been multiple publications verifying the critical role of ACE2 in mediating the virus–host interaction and several groups have developed surrogate neutralization assays (ELISA and CLIA) based on the interaction between the viral spike protein and its receptor ACE2 [5,6].

In this study, we sought to replicate an FDA/EUA-approved ELISA-based serology assay developed at Mount Sinai, New York [7] and, with minor modification to the assay protocol, build in the capacity to identify antibodies capable of blocking the interaction with ACE2. Such antibodies would be neutralizing and would be capable of blocking the entry of the virus into the host cell; however, antibodies that bind the N-terminal domain (NTD) have also been identified as having neutralizing activity and can block in viral infectivity assays [8]. We tested pre-COVID-19 and SARS-CoV-2-infected sera in the serology assay and showed a good correlation of our test results with those generated by the Krammer group. Testing of a subset of the infected patient sera in the integrated ACE-2 binding assay correlated closely with a spike-based pseudovirus assay which was run in parallel.

## 2. Materials and Methods

### 2.1. Recombinant Proteins

**Figure 1:**
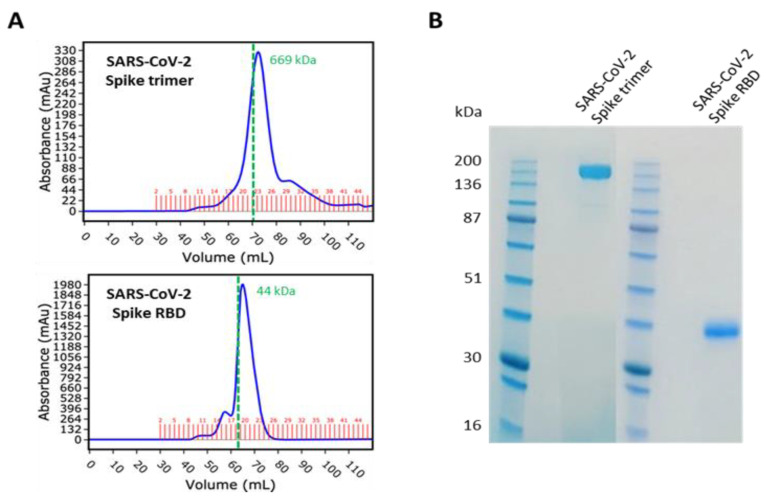
SARS-CoV-2 spike and RBD expression and purification by Peak Proteins Ltd. (**A**) Size exclusion chromatography UV absorbance trace of purified recombinant SARS-CoV-2 spike trimer (top, Superose 6 16/60 column, fractions 19–26 pooled) and SARS-CoV-2 spike RBD (bottom, Superdex 75 16/60 column, fractions 18–25 pooled). Retention volume of molecular weight standard thyroglobulin (top) and ovalbumin (bottom) marked as dashed green lines. (**B**) Reducing SDS-PAGE analysis of purified recombinant spike proteins.

The SARS-CoV-2 spike trimer (aa14–1213) and RBD (aa319–541), both with a C-terminal 6His tag, were expressed and purified by Peak Proteins Ltd., Macclesfield, UK (www.peakproteins.com, accessed 13 July 2021), along with human ACE2 ECD (aa19–615) with a C-terminal Avi tag. The proteins were produced in HEK293T cells. The spike proteins were purified by nickel affinity chromatography, followed by size exclusion chromatography (Figure 1). The ACE protein was purified by anion exchange chromatography, followed by size exclusion chromatography. Concentration was determined by A280 correcting for extinction coefficient.

### 2.2. Human Samples

Human serum samples were obtained from the St James’s, Tallaght University Hospital, TCD Allied Research (STAR) Bioresource. Antibody serum data for the Abbott and Roche antibody kits were also provided through this resource.

### 2.3. ELISA

The ELISA protocol was based on a published method from the Icahn School of Medicine at Mount Sinai, New York [7]. Briefly, 96-well plates (Greiner Bio-one, Gloucestershire, UK) were coated with 50 µL of 2 µg/mL SARS-CoV-2 RBD or full-length spike trimer in PBS (Gibco; Thermo Fisher Scientific, Waltham, MA, USA) and left overnight at 4 °C. The following morning, the coating solution was removed and 100 µL of 3% non-fat milk, prepared in PBS with 0.1% Tween 20 (PBST), was added to each well as blocking buffer. The blocking solution was removed, and the serum samples were diluted 1:50 in 1% milk PBST. After dilution, 100 µL of the diluted samples were added to wells in triplicate and left for 2 h at room temperature. The plates were then washed three times with 200 µL of 0.1% PBST. The HRP-conjugated secondary antibodies were diluted in 1% milk PBST and 100 µL/well was added for 1 h at room temperature; IgG1 (1:2000, Southern Biotech, Birmingham, AL, USA, #9054-05), IgM (1:3000, (Sigma-Aldrich, St Louis, MO, USA, #A6907-1ML), IgA (1:3000, (Sigma-Aldrich, St Louis, MO, USA, #A0295-1ML). The antibody solution was removed, and the plates were again washed three times with 0.1% PBST. After washing, 100 µL SigmaFast OPD (o-phenylenediamine dihydrochloride, Sigma-Aldrich, St Louis, MO, USA, #P9187) was added to each well for 10 min and the reaction was stopped using 3M hydrochloric acid. The optical density was measured at 492 nm using a Multiskan FC (Thermo Fisher Scientific, Waltham, MA, USA) plate reader and values were imported into GraphPad Prism 8 for analysis. Cut-off values were determined by averaging the OD values for all pre-COVID-19 samples plus the addition three standard deviations. All ELISAs were performed in a COVID-19-dedicated room under BSL-2 conditions or above.

### 2.4. In Vitro ACE2 Binding Assay

As before, 96-well plates were coated with RBD and blocked. Serum samples were prepared in 1% milk PBST, serially diluted two-fold from a 1:50 dilution and left on the plates for 1 h at room temperature. A positive control was included in the form of a commercial IgG1 anti-spike antibody containing 2% normal human serum and diluted as previously described (Thermo Fisher Scientific, Waltham, MA, USA, MA5-35939). After washing three times with 0.1% PBST, 50 µL/well of 10 µg/mL biotinylated ACE2 was added to the plates and left for 1 h at room temperature. The ACE2 solution was removed and the plates washed three times. Streptavidin HRP was diluted 1:40 in PBS and 100 µL/well was added to the plate for 20 min. The streptavidin HRP was removed and the plates were washed three times. After washing, 100 µL SigmaFast OPD was added to each well for 10 min and the reaction was stopped using 3M hydrochloric acid. The optical density was measured at 492 nm using a Multiskan FC plate reader. The OD values were then directly imported into GraphPad Prism 8 for analysis.

### 2.5. Pseudovirus Infection Assay

Lentiviral-based pseudoparticles were generated in HEK293T cells based on a scaled-up version of a previously published method [9]. HEK293T cells were seeded at 4 × 106 cells in 10 cm dishes for 24 h at 37 °C in 5% CO_2_ prior to infection. The cells were then transfected with plasmid DNA using Gene Juice (Merck Millipore, Darmstadt, Germany, #70967) as follows: 3.55 µg P8.91 (encoding HIV-1 gag-pol), 3.55 µg CSFLW (lentivirus backbone expressing a firefly luciferase reporter gene), and 150 ng of pCAGGS SARS-CoV-2 spike. The transfected cells were incubated for 24 h and the medium was replaced. Supernatants were then harvested at 48 and 72 h post-transfection and pooled. Pooled supernatants were then filtered (0.2 µm) to remove cellular debris and frozen at −80 °C.

One day prior to the assay, HEK-Blue hACE2 cells (Invivogen, Toulouse, France, hkb-hace2) were seeded with 100 µL of Dulbecco’s Modified Eagle’s medium (DMEM) in 96-well plates at 2 × 10^4^ cells/well. After 24 h, serum from COVID-19 patients was serially diluted two-fold from a 1:50 dilution and mixed with a 1:4 dilution of pseudovirus particles (based on previous titration). One hundred microliters of this was transferred onto the cells and incubated for 72 h at 37 °C. The medium was removed, and the cells were lysed in passive lysis buffer. The lysates were subsequently analyzed for luciferase activity on a Thermo Scientific Luminoskan Microplate Luminometer with Ascent software v2.6 and imported into GraphPad Prism 8 for analysis.

### 2.6. Statistical Analysis

Statistical analysis was performed using GraphPad Prism 8 version 8.0.2 and statistical significance was determined between samples using the Mann–Whitney U test.

## 3. Results

### 3.1. A SARS-CoV-2 Serological Assay Based on the Spike Protein

**Figure 2:**
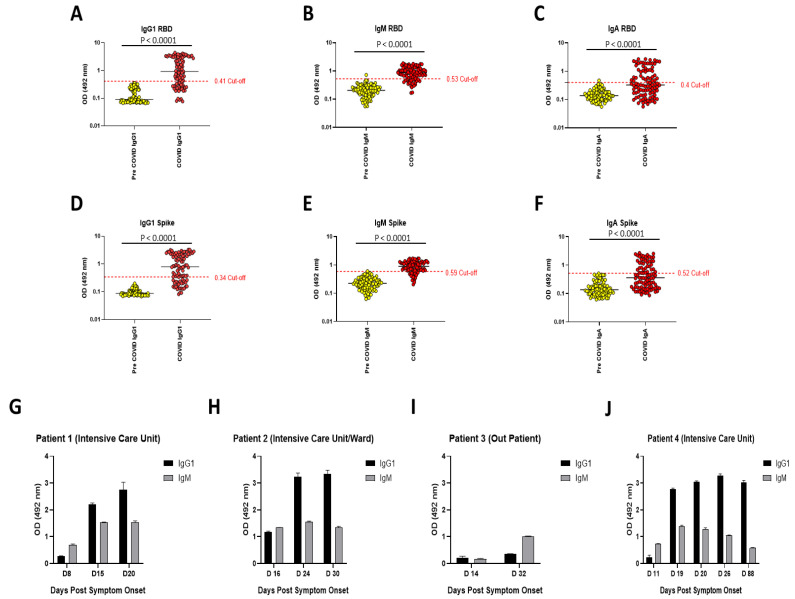
OD values representative of antibody reactivity in sera from pre-COVID-19 and confirmed SARS-CoV-2 patients. (**A**–**C**) Results of samples tested against RBD. (**D**–**F**) Results of samples tested against full-length spike protein. Statistical analysis was performed using Mann–Whitney U tests in GraphPad Prism. The samples represented here were taken >7 days post symptom onset and include 103 pre-COVID-19 and 91 SARS-CoV-2 positive samples. Horizontal lines represent mean values and samples were performed in triplicate. (**G**–**J**) Examples of results from longitudinal samples.

Since the beginning of the SARS-CoV-2 pandemic, the global research effort has placed a high priority on the development of effective serological assays to detect previous exposure and immunity to the virus. We replicated the assay published by Amanat et al. [7], with minor modifications. These modifications include not employing serum serial dilutions to measure antibody reactivity, the use of IgG1 as a marker for IgG humoral responses against SARS-CoV-2 and the integration of an in vitro ACE2 binding assay into the protocol. In total, we tested 103 pre-COVID-19 samples and 91 samples from patients with confirmed SARS-CoV-2 infection. Figure 1 shows the purity of the spike proteins used in the study (full length trimeric spike protein and the receptor binding domain or RBD). Both versions of the spike protein migrate as expected on size exclusion chromatography and are highly pure when analyzed on reducing sodium dodecyl sulphate-polyacrylamide gel electrophoresis (SDS-PAGE). Figure 2A–F shows the reactivity of sera against RBD and full-length spike protein in pre-COVID-19 and COVID-19 patient serum samples taken >7 and <104 days post symptom onset. The median age was 53 years with 45 samples from males and 46 from females (Table S1). The pre-COVID-19 and COVID-19 patient samples were obtained from the St James’s, Tallaght University Hospital, Trinity Allied Research (STTAR) Bioresource, with disease severity ranging from mild to severe. Optical density (OD) values representative of immunoglobulin levels in the cohorts were obtained for three relevant isotypes: IgG1, IgM and IgA. A cut-off value for each isotype was derived from the mean value of the pre-COVID-19 samples, along with the addition of three standard deviations. Samples exceeding the cut-off were considered positive. We obtained a sensitivity and specificity of 88.5% (95%CI 79.5–93.81) and 99.1% (95%CI 94.8–99.95) respectively, for combined IgG1 and IgM results. This is marginally lower than the 95% sensitivity and 100% specificity reported in Stadbauer et al., which utilized the Krammer serology assay [10]. As evident in the longitudinal samples (Figure 2G–J), the stage of infection plays an important role in the detection of anti-spike antibody isotypes in COVID-19 patients. This is also consistent with the kinetics of antibody responses during SARS-CoV-2 infection [11].

**Figure 3:**
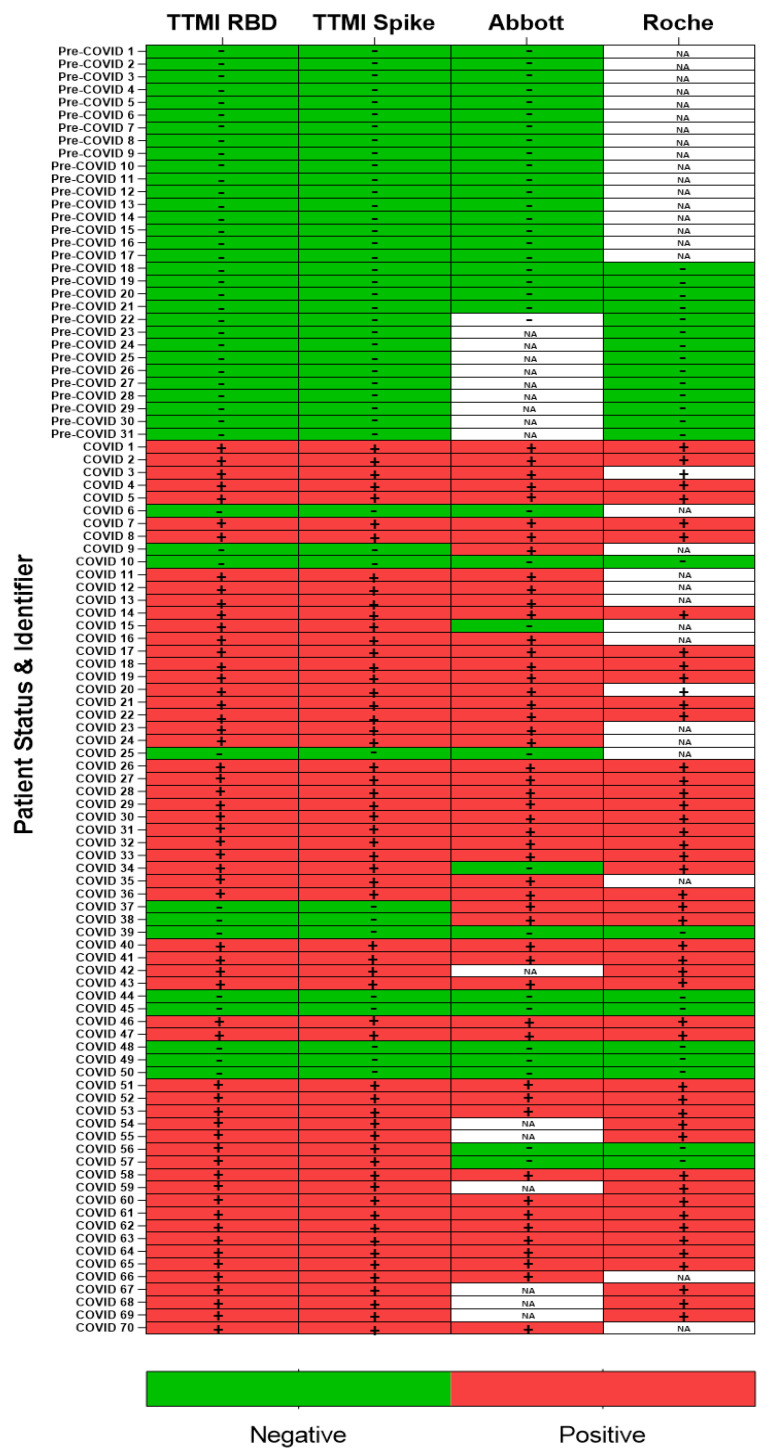
Comparison between the SARS-CoV-2 anti-spike serology assay and commercially available assays. Samples tested with the RBD and spike proteins are shown as independent assays and are comprised of IgG1 and IgM patient data. These were compared with the same samples tested by the Roche and/or the Abbott assays where *n* = 69 and *n* = 84, respectively. Samples indicative of seropositivity are shown as “+”and negative as “−“. The tiles containing “NA” indicate samples in which no data is available for that assay.

We analysed samples that were tested by commercial SARS-CoV-2 antibody serology assays (Roche Elecsys Anti-SARS-CoV-2 and Abbott SARS-CoV-2 IgG anti-N protein assays) and compared our results for IgG1 and IgM (Figure 3 and Table 1). Detection of either isotype was considered a ‘positive’ result for the sample. For the Roche assay, the cut-off index for a positive result was ≥1.0 and for the Abbot assay, the cut-off index for a positive result was ≥1.4. According to manufacturer websites, the assay sensitivities and specificities are reported to be 99.5% and 99.5%, respectively, at ≥14 days for the Roche assay, and 100% and 99.63%, respectively, at ≥14 days for the Abbott assay [12,13]. However, independent evaluations by Public Health England have reported a sensitivity of 86.1% and specificity of 100% for the Roche assay, and a sensitivity of 93.9% and specificity of 100% for the Abbott assay [14,15]. We demonstrated that the Krammer serology assay is comparable to the widely used and approved Roche and Abbott N-protein assays in terms of both sensitivity and specificity with concordances of 94.2% and 91.7% respectively.

### 3.2. In Vitro ACE2 Binding Assay

**Figure 4:**
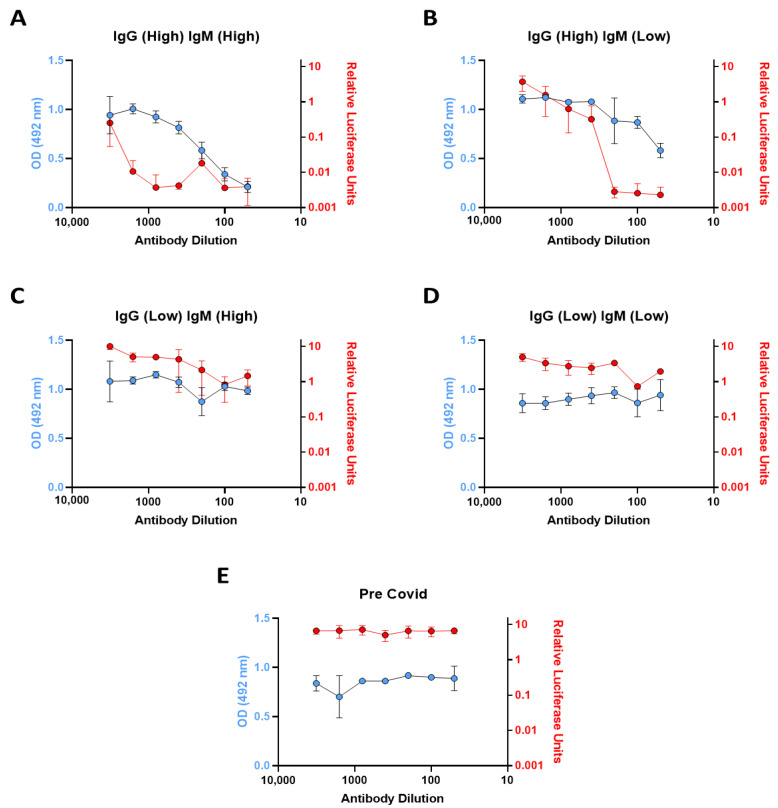
Antibody spike RBD neutralization capacity in patient sera with different immunoglobulin profiles. (**A**–**E**) Demonstrates the spike RBD neutralization capacity of patients with various immunoglobulin profiles using serially diluted serum. The blue points represent the data generated using the in vitro ACE2 binding assay and the red points represent data generated from the same sample using the pseudovirus assay. Data are the mean ± the standard deviation of triplicate samples from a representative experiment (*n* = 3).

In order to investigate the neutralization capacity in selected patient samples, we integrated an in vitro ACE2 binding assay into the serology protocol. We performed the in vitro ACE2 binding assay using patient serum samples, which we categorized into four different immunoglobulin profiles by serology assay screening; (High IgG, High IgM), (High IgG, low IgM), (Low IgG, High IgM), and (low IgG, low IgM). Individual patient samples from each category were distinguished as high and low for each isotype based on the ratio to the cut-off value and the samples were taken 21–88 days post symptom onset (*n* = 3). A sample was considered to have a high reactivity for IgM if it was ≥3× the cut-off value or a high reactivity for IgG if it was ≥7× the cut-off value. Samples for both isotypes were considered to have a low reactivity if they were positive but <2× the cut-off value. These values are based on the maximum and minimum antibody reactivity for each isotype obtained during the prior serology analysis. From the serology, the portion of patient samples in these categories are as follows: 6.59% High IgG, High IgM; 14.3% High IgG, Low IgM; 4.4% Low IgG, High IgM; 5.5% Low IgG, Low IgM. In the patients considered to have high IgG1 and high IgM antibody reactivity, a 1 in 50 dilution of serum results in 80% reduction in the signal indicating potent neutralization (Figure 4A). While the High IgG1, Low IgM samples also possessed potent ACE2-binding neutralization capacity, particularly at lower dilutions, these were not as effective as the former (Figure 4B). Patients with low IgG and high IgM, along with low IgG and low IgM profiles, exhibited poor ACE2-binding neutralization capacity in comparison with the first two categories (Figure 4C,D). These patterns, as expected, were similar in two additional patient samples from these immunoglobulin profile categories. To correlate the activity in the ACE2-RBD binding assay we employed a pseudovirus neutralization assay derived from the previously published method [9], to test those sera. This system involves measuring the infection of stable ACE2-expressing HEK293T cells (Invivogen) with pseudovirus containing the SARS-CoV-2 spike protein, which expresses firefly luciferase on successful infection. Neutralizing capacity of post-infection sera can therefore be estimated through a decrease in luciferase activity when compared to control sera. Similar profiles were generated using this pseudovirus system shown in red, when compared to the in vitro ACE2 binding assay results shown in blue (Figure 4A–E). This indicates a consistent effect and a reasonable correlation between the in vitro ACE2 binding assay and the cell-based pseudovirus infection assay, which was expected given the pseudovirus system, while more complex, is also primarily based on the spike-ACE2 interaction, though in a pseudoviral cell infection model.

## 4. Discussion

Our understanding of SARS-CoV-2 immunity is continuously improving, particularly in terms of longevity of antibody responses and efficacy of antibody neutralization post-vaccination. SARS-CoV-2 serology testing has a number of qualities that make it a vital tool in the ongoing pandemic and its aftermath. In addition to measuring antibody responses in both infection and vaccination, the modified assay format we report can also be used to screen for potential donors for convalescent plasma therapy and evaluate their levels of neutralizing antibodies.

We replicated the SARS-CoV-2 serology assay developed at the Icahn School of Medicine, New York. On the 15 April 2020, this assay was given emergency use authorization by the FDA [7]. The assay involves the detection of anti-RBD IgG antibodies with subsequent confirmatory detection of antibodies against full-length spike protein. The primary advantage of this test is its relative low cost compared with other commercial immunoassays for SARS-CoV-2 antibodies. However, it also offers an advantage in a research setting as it can be used to distinguish between antibody isotypes by simply using isotype-specific horseradish peroxidase (HRP)-conjugated secondary antibodies [7]. This assay is particularly attractive in a research setting, not only due to cost efficiency but also because of the ability to format it into a neutralization assay with few modifications. Locally, to run an IgG1 test, materials cost approximately €0.50/$0.61/£0.43 per sample, taking into consideration the cost of the coating protein, substrate, the ELISA plate, control and secondary HRP-conjugated antibodies, and phosphate buffered saline (PBS).

The work presented herein replicates a highly sensitive and specific (88.5% and 99.1% respectively, for combined IgG1 and IgM results) SARS-CoV-2 serology assay. We obtained sensitivities of approximately 70% for IgG1 and 77% for IgM, respectively. Conversely, IgA appears to be a more sporadic indicator of immunity as the sensitivity was only approximately 43%. However, this result is unsurprising in some ways as the assay is serum-based and IgA tends to be associated with immunity at mucosal surfaces. However, this may just represent a lack of research on this isotype during SARS-CoV-2 infection [16]. Thus, investigating IgA in saliva samples may be a more appropriate approach. The longitudinal samples demonstrate the ability of the assay to track dynamics of isotype-specific changes over the course of infection. In three out of the four examples, with the first sample taken 8–14 days post symptom onset, antibody reactivity for IgG1 was below the cut-off point for a positive result. However, with the exception of patient 3, all of these samples were positive for IgM. In all four patients, antibody reactivity then proceeded to increase in accordance with known antibody trends. A recent study shows that antibody levels in COVID-19 patients can take up to 22 days to become detectable with most samples having detectable levels >14 days post symptom onset [11]. IgM tends to peak at 15–35 days post symptom onset and then decline, whereas IgG plateaus after 22–35 days post symptom onset before declining at a much later stage. Therefore, the variation in antibody induction time we see here is not a novel observation. Interestingly, using the Krammer assay, Stadlbauer et al. obtained a sensitivity of 95% and specificity of 100% for IgG [10]. However, we chose an IgG1-specific secondary due to the prominence of IgG1 as a marker for an effective IgG humoral response against SARS-CoV-2 [17,18,19,20]. Furthermore, we investigated IgM in parallel to IgG1 at >7 days post symptom onset. As the Stadlbauer et al. calculations were based on a smaller sample size, and that it can take >14 days for IgM to become detectable, which in most cases precedes IgG induction, it is likely that our early sampling, along with the use of the IgG1 secondary antibody, is responsible for this discrepancy.

We compared the Krammer assay with two commercially available antibody assays from Roche and Abbott that target the viral N protein. The Roche assay is an electrochemiluminescence immunoassay (ECLIA) and requires a Roche e411 analyzer. The Abbott assay is a two-step chemiluminescent microparticle immunoassay (CMIA) intended for the qualitative detection of IgG and requires an Abbott Architect i4000sr analyzer. Of the samples we obtained from the STTAR Bioresource, we identified 70 COVID-19 positive and 31 pre-COVID-19 samples that were tested using one or both platforms, and these demonstrate that our results are largely consistent across the other two assays for confirmed COVID-19 positive and pre-COVID-19 samples (*n* = 101). While the commercial platforms have the advantage of speed due to automation with mix and read format, of particular importance in a clinical setting, the assay used here has advantages when it comes to the ability to distinguish between antibody isotypes at a low cost while also maintaining analogous sensitivity and specificity.

In addition to replicating the Krammer serology assay, demonstrating its robust utility and comparability to other approved commercial assays, we re-formatted the assay to develop an assay capable of screening for neutralizing spike-ACE2 anti-spike antibodies. While serological assays are key tools for tracking SARS-CoV-2 seroconversion and for investigating the effectiveness of vaccinations, they do not give insights into antibody neutralization capacity which provides a more effective indication of humoral protection. It has become clear that neutralizing antibody levels are a key factor in recovery and potential immunity to SARS-CoV-2 [21,22]. As such, we sought to integrate an element of antibody neutralization assay into the existing serology assay format with only minor modification of the serology assay protocol. The basic serology protocol steps remain the same, but in place of an isotype-specific secondary antibody to detect bound antibodies, biotinylated ACE2 was used to determine the presence or absence of neutralizing antibodies which block the interaction of spike RBD to ACE2. We recognize that this ACE2-RBD binding assay will not detect all types of neutralizing antibodies, only those capable of blocking the spike-ACE2 interaction. For example, antibodies against the NTD that are neutralizing would not be detected in this assay [8].

Our analysis of neutralization capacity assayed four types of patient sample representative of different immunoglobulin profiles based on the serology results. The High IgG, High IgM and High IgG, Low IgM were, as suspected, the most efficient at ACE2-spike neutralizing capacity. Interestingly, the three Low IgG, High IgM samples demonstrated poor neutralization ability, and this may be indicative of an early low affinity IgM response. We subsequently tested these samples using a spike-based pseudovirus infection assay and demonstrated similar profiles to those of the in vitro ACE2 binding assay. While both methods are dynamically different in terms of interaction with the SARS-CoV-2 spike protein, along with the requirement of viral entry in the pseudovirus infection assay, consistency between these two assays indicate that the recombinant ACE2-RBD binding assay is a fast and convenient novel method to evaluate the neutralization capacity of anti-SARS-CoV-2 antibodies. The ability to perform ACE2-spike neutralization assays within this system offers a valuable modular tool for future research.

## 5. Conclusions

As the COVID-19 pandemic continues to take an enormous toll on human health, mortality and economies throughout the world, scientific advances such as newly available vaccines offer a hopeful route out of this pandemic. With vaccines beginning to rollout globally, serology testing will come to the forefront of research, providing us with valuable information regarding vaccine efficacy and longevity in the wider population. Here we have replicated a versatile and cost-effective alternative ELISA-based serology assay to those commercially available. Using this assay, we have also integrated an element of antibody neutralization into the protocol which can determine the neutralizing capacity of antibodies that block the ACE2-spike interaction in sera from patients with different immunoglobulin profiles. While antibody responses represent only one arm of the immune response against SARS-CoV-2, these tools provide an affordable and versatile option for future research in this area.

## Figures and Tables

**Table 1:** Comparison between the SARS-CoV-2 ELISA and the commercial assays using a two-tailed Fisher’s exact test.

Test	Positive	Negative	N	Odds Ratio	95% CI	*p*
Abbott	50	34	84	0.9516	0.5226 to 1.729	>0.9999
RBD/Spike	51	33	84			
Roche	46	23	69	1.000	0.4840 to 2.066	>0.9999
RBD/Spike	46	23	69			

## Data Availability

The data that support the findings of this study are available from the corresponding author upon reasonable request.

## Supplementary Materials

The following are available online at https://www.mdpi.com/article/10.3390/v13071371/s1, Table S1: Summary of clinical information on SARS-CoV-2 positive patients.
