# Supplementary material for: Dynamic Assay for Profiling Anti-SARS-CoV-2 Antibodies and Their ACE2/Spike RBD Neutralization Capacity
Source: Viruses. 2021 Jul 15;13(7):1371. doi: 10.3390/v13071371 (PMC8309970; doi:10.3390/v13071371)
Supplement: Supplementary file 1 [file viruses-13-01371-s001.zip › viruses-1277166-si final.pdf]

Table S1. Summary of clinical information on SARS-CoV-2 positive patients

Variable		N=(91)	(%)
**Sex**	Male	45	49.5
	Female	46	50.5
**Age**	Median	53	
	Range	19-84	
**Disease Severity**	Mild	46	50.5
	Moderate	13	14.3
	Severe	32	35.2
